# Charakterisierung von Sonnenschutzleistung: Quo vadis?

**DOI:** 10.1007/s00105-022-04958-x

**Published:** 2022-03-25

**Authors:** Uli Osterwalder, Christian Surber

**Affiliations:** 1 Sun Protection Facilitator GmbH, Pfeffingerstr. 82, 4053 Basel, Schweiz; 2 Dermatologische Klinik, UniversitätsSpital Zürich, Gloriastr. 31, 8091 Zürich, Schweiz; 3 Dermatologische Klinik, Universitätsspital Basel, Petersgraben 4, 4031 Basel, Schweiz

**Keywords:** Sonnenschutzmittel, Lichtschutzfaktor, Strahlungsspektrum, Hybride diffuse Reflexionsspektroskopie, Spektrale Homöostase, Sunscreening agents, Sun protection factor, Radiation spectrum, Hybrid diffuse reflectance spectroscopy, Spectral homeostasis

## Abstract

Die Aufgabe der ersten Sonnenschutzmittel war es, die Entstehung von Sonnenbrand zu verhindern und, dem Zeitgeist der 1950/60er-Jahre folgend, die Bräunung der Haut nicht zu beeinträchtigen. Schnell entstand die Notwendigkeit, die Schutzleistung zu quantifizieren. Ursprünglich unter Zuhilfenahme des natürlichen – heute eines künstlichen – Sonnenlichts wurde eine Methode zur Bestimmung eines Sonnenschutzfaktor (SPF) entwickelt. Dieser ist heute formal als das Verhältnis zwischen minimaler erythemwirksamer UV-Dosis auf mit Sonnenschutzmittel geschützter und minimaler erythemwirksamer UV-Dosis auf ungeschützter Haut definiert (ISO 24444:2019). Drei Beobachtungen stellen die Eignung der Methode infrage: 1) Zwischen-Labor-Variabilität: Trotz strenger Normierung sind Resultate von SPF-Bestimmungen aus verschiedenen Labors und Regionen sehr großen Schwankungen unterworfen. 2) Natürliches vs. künstliches Sonnenlicht: Das Strahlungsspektrum des künstlichen Sonnenlichts unterscheidet sich von dem des natürlichen Sonnenlichts. Die mit künstlichem Sonnenlicht bestimmten SPFs (wie auf allen derzeit im Handel befindlichen Sonnenschutzmitteln abgebildet) sind im Vergleich zur SPF-Bestimmung mit natürlichem Sonnenlicht deutlich zu hoch. 3) Erythembelastung: Bei der Bestimmung des SPF werden die Probanden potenziell schädlicher Strahlung ausgesetzt. Vor diesem Hintergrund werden alternative Methoden – In-vitro-SPF, hybride diffuse Reflexionsspektroskopie (HDRS) und In-silico-Berechnungen – vorgestellt. Diese haben das Potenzial, die heutige mit erheblichen Einschränkungen verbundene Methode abzulösen. Als Sofortmaßnahme wird die Rückbesinnung auf die für alle verständliche Beschreibung *niedriger*, *mittlerer*, *hoher* und *sehr hoher* Schutz empfohlen, in Zukunft unter Berücksichtigung des Spektrums des natürlichen Sonnenlichtes.

## Abstract (en)

The task of the first sunscreens was to prevent the development of sunburn and, following the spirit of the 1950/1960s, to not impair the tanning of the skin. The need to quantify the protective performance soon arose. Originally with the help of natural—nowadays artificial—sunlight, a method was developed to determine a sun protection factor (SPF). It is formally defined as a ratio between minimum erythema-effective UV dose on sunscreen-protected skin and minimum erythema-effective UV dose on *unprotected* skin (ISO 24444:2019). Three observations question the suitability of the method. (1) Interlaboratory variability: Despite strict standardization, results of SPF determinations from different laboratories are subject to large variations. (2) Natural vs. artificial sunlight: The radiation spectrum of artificial sunlight differs from that of natural sunlight. SPFs determined with artificial sunlight (as depicted on all sunscreens currently on the market) are significantly too high compared to SPF determination with natural sunlight. (3) Erythema burden: When determining SPF, subjects are exposed to potentially harmful radiation. Against this background alternative methods—in vitro SPF, hybrid diffuse reflectance spectroscopy (HDRS) and in silico calculations—are presented. These have the potential to replace the current method. As an immediate measure, it is recommended to return to the comprehensible description of low, medium, high, and very high protection and in the future to take into account the spectrum of natural sunlight.

Sonnenschutzmittel sind ein wichtiges Mittel zur Prävention von Sonnenbrand, Hautkrebs und Hautalterung. Die Leistungsfähigkeit dieser Produkte wird manchmal angezweifelt, sei es aufgrund von widersprüchlichen Ergebnissen bei Nachtests des Sonnenschutzfaktors (SPF) oder Selbsterfahrung mit Sonnenbrand bei Verwendung von Produkten mit hohem SPF. Die selektive Schutzerfassung gängiger Methoden (SPF, UVA-PF [„ultraviolet A protection factor“]) sind mögliche Gründe für diese Beobachtung. Alternative Methoden erlauben, den Schutz monochromatisch – Wellenlänge für Wellenlänge – zu eruieren. Damit kann das Schutzprofil eines Sonnenschutzmittels mit einem idealen Schutzprofil, bei dem das gesamte Spektrum des Sonnenlichts reduziert wird, verglichen werden.

Die zentrale Aufgabe der ersten Sonnenschutzmittel war es, die Entstehung von Erythemen zu verhindern und, dem Zeitgeist der 1950/60er-Jahre folgend, die Bräunung der Haut nicht zu beeinträchtigen. Mit der Einführung von Sonnenschutzmitteln entstand schnell auch die Notwendigkeit, die Schutzleistung zu quantifizieren. Rudolf Schulze veröffentlichte 1956 in der deutschen Zeitschrift *Parfümerie und Kosmetik* ein Verfahren zur Bestimmung der Schutzleistung von Sonnenschutzmitteln am Menschen [32]. Der Sonnenschutzfaktor (SPF) wurde definiert als ein Maß dafür, wie viel natürliches Sonnenlicht erforderlich ist, um ein Erythem auf geschützter Haut (d. h., in Gegenwart von Sonnenschutzmittel) im Verhältnis zu ungeschützter Haut zu erzeugen. Aus praktischen Gründen wurde die zunächst verwendete natürliche Sonnenstrahlung bald durch eine künstliche Strahlungsquelle ersetzt. Unterdessen sind die formale Definition (SPF = minimale erythemwirksame UV-Dosis geschützt/minimale erythemwirksame UV-Dosis ungeschützt) und das Messverfahren als Goldstandard in einer internationalen Norm definiert (ISO 24444:2019, FDA-Rule) [13, 20]. Wie ursprünglich angedacht, war es v. a. die erythemwirksamere UVB-Strahlung (Sonnenbrand), vor der man sich schützen wollte. Die UVA-Strahlung hingegen war erwünscht, um die Bräunung der Haut nicht zu behindern. Es ist daher nicht verwunderlich, dass der SPF oft primär mit dem UVB-Schutz in Verbindung gebracht wird.

UVA-Strahlung war erwünscht, um die Bräunung nicht zu behindern

Unterdessen ist jedoch belegt, dass auch UVA-Strahlung zur Erythembildung und schließlich zur Bildung von Hautkrebs beiträgt. Vor diesem Hintergrund wurde in den 1990er-Jahren – in Ergänzung zum SPF_ISO24444_ – ein In-vivo-Test entwickelt, der spezifisch über den UVA-Schutz eines Produkts Auskunft gibt. Das Surrogat in dieser Testung ist die minimale UVA-Dosis zur Erzeugung einer PPD(„persistent pigment darkening“)-Reaktion in der menschlichen Haut. Die PPD-Reaktion wird durch die Oxidation des Melanins in der Haut verursacht und als Melaninbräune sichtbar. Die Bestimmung der PPD-Reaktion ist ebenfalls international normiert (ISO 24442:2011) [18]. Für die PPD-Reaktion gibt es kein klinisches Korrelat wie beim SPF_ISO24444_ mit dem Erythem. Als weitere Ergänzung wurde auch eine In-vitro-Methode zur Quantifizierung des UVA-Schutzes (ISO 24443:2012) entwickelt [19]. Nach einem verbreiteten Verständnis ist ein angemessener Schutz im UVA-Bereich dann gegeben, wenn das Verhältnis UVA-PF_ISO24443_ oder UVA-PF_ISO24442_ zu SPF_ISO24444_ gleich oder größer als 1/3 ist (Artikel 15 der Empfehlung, [5]). Diese Information – dargestellt als Signet (UVA in einem Kreis) – sollte es dem Verbraucher ermöglichen, Produkte zu wählen, die einen ausreichenden Schutz im UVA-Bereich bieten [3, 29]. Der SPF, aber auch das UVA-Signet gelten heute als Minimalanforderung an ein Sonnenschutzmittel. Um den Produkten Alleinstellungsmerkmale zu verleihen, wurden weitere Merkmale entwickelt, um besondere Schutzleistungen hervorzuheben. Dazu gehört der Schutz vor Blau- oder sichtbarem Licht bis hin zu Infrarotlicht und weiteren Schutz-Claims [33]. Für die Bestimmung dieser Schutzleistungen gibt es keine normierten Verfahren. Im Gegensatz zu den Verfahren zur Bestimmung des SPF oder UVA-PF, wo der Schutz als Folge der Absorption von Strahlung durch UV-Filter *auf der Haut* bestimmt wird, erfolgt beim Schutz vor Blau- oder sichtbarem Licht bis hin zu Infrarotlicht keine Absorption von Strahlung durch Filter, sondern strahlungsbedingt entstandene schädliche Agenzien (z. B. freie Radikale) *in der Haut* werden beispielsweise durch die Beigabe von Antioxidanzien neutralisiert.

Im folgenden Beitrag berichten die Autoren über die gegenwärtigen Erfahrungen und Erkenntnisse mit den normierten Methoden und über mögliche Weiterentwicklungen.

## SPF_ISO24444_ und weitere Verfahren

Aufgrund breiter Anwendung des SPF_ISO24444_-Messverfahrens sind 3 Beobachtungen in den Mittelpunkt der wissenschaftlichen Diskussion getreten.

### Zwischen-Labor-Variabilität.

Miksa et al. zeigten im Rahmen eines großen Ringversuches, dass trotz strenger Normierung Resultate aus verschiedenen Labors sehr großen Schwankungen unterworfen sind. Sie empfehlen deshalb, Resultate aus 3 bis 4 verschiedenen Labors zusammenzutragen, um einen zuverlässigen Wert zu erhalten [26]. Da in der Praxis häufig nur eine Messung in einem Labor durchgeführt wird, stellt diese Beobachtung sowohl die SPF-Deklaration auf den Produktpackungen wie auch die Verdikte über die SPF-Deklarationen von Konsumentenschutzorganisationen infrage. Darüber hinaus ist die Durchführung solcher Messungen in 3 bis 4 verschiedenen Labors sehr kostenintensiv. Da die Bestimmung der Wasserfestigkeit ebenfalls auf der SPF_ISO24444_-Messung beruht, gelten auch hier die Einschränkung in Bezug auf die Zuverlässigkeit der Messung. Ob diese Beobachtung auch für andere Bestimmungsmethoden wie UVA-PF_ISO24443_ oder UVA-PF_ISO24442_ zutrifft, ist nicht bekannt bzw. nicht publiziert.

### Natürliches versus künstliches Sonnenlicht.

**Figure 1:**
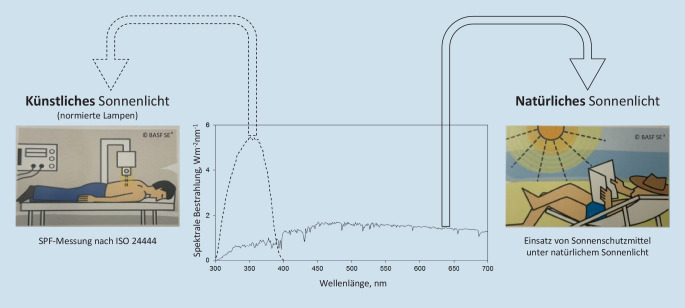

**Figure 2:**
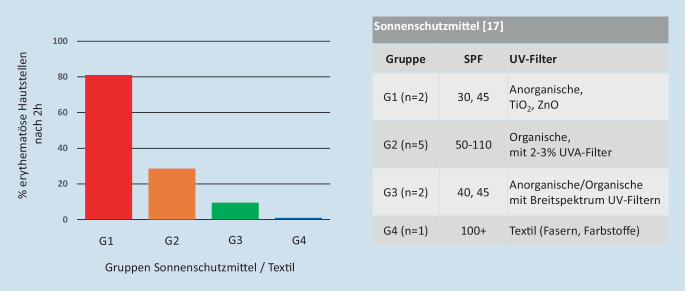

Die ursprünglichen Bestimmungen des SPF erfolgten im Freien mit natürlichem Sonnenlicht. Aus praktischen Gründen wurde die natürliche Lichtquelle durch eine normierte Lampe ersetzt (Annex B, [20]). Allerdings verlor sich dabei das Bewusstsein, dass das Strahlungsspektrum der Lampe sich vom natürlichen Sonnenlicht deutlich unterscheidet. Die Abb. 1 zeigt deutlich, dass der Strahlungsbereich der Lampe v. a. in der UV-Region (290–400 nm) liegt. Vor Kurzem haben Diffey und Osterwalder im Rahmen von theoretischen Überlegungen darauf hingewiesen, dass aufgrund des Fehlens des gesamten Strahlungsspektrums die bestimmten SPF_ISO24444_-Werte zu hoch sind [8]. Sie äußerten die Vermutung, dass Produkte mit der Kennzeichnung SPF50+ möglicherweise nicht mehr als ein SPF25 an Schutz gegen das gesamte Sonnenlichtspektrum leisten. Gestützt auf experimentelle Arbeiten, haben bereits davor 2 Forschungsgruppen auf dieses Phänomen hingewiesen. Die Publikationen wurden allerdings kaum beachtet [2, 23]. Hughes und Granger untermauern die früheren Arbeiten sowie die Vermutung von Diffey und Osterwalder mit neueren In-vivo-Untersuchungen am Menschen mit natürlichem Sonnenlicht (Abb. 2; [15, 17]).

### Erythembelastung.

Bei der Bestimmung des SPF_ISO24444_ und des UVA-PF_ISO24442_ werden die Probanden potenziell schädlicher Strahlungsmenge ausgesetzt. Vor diesem Hintergrund ist es naheliegend, diese Methoden durch In-vitro- oder nichtinvasive In-vivo-Bestimmungen zu ersetzen.

Die oben genannten Beobachtungen sind vielen noch wenig bewusst, und v. a. wurden noch keine Empfehlungen verfasst, wie mit diesen Beobachtungen umzugehen ist.

### Zwischen-Labor-Variabilität.

Die Zwischen-Labor-Variabilität ist in verschiedenen Disziplinen einschließlich Labormedizin ein bekanntes Phänomen, dem man Beachtung schenken muss und das man mit Ringversuchen zu charakterisieren versucht [35]. Erst mit der Veröffentlichung der Miksa-Untersuchungen wurde dieses Phänomen im Bereich der Sonnenschutzcharakterisierung allgemein bekannt [26]. Um der Zwischen-Labor-Variabilität begegnen zu können, ist als Sofortmaßnahme nur die konsequente Durchführung mehrerer Messungen in verschiedenen Labors zu empfehlen. Dies gilt sowohl für die Charakterisierung des Sonnenschutzmittels durch den Hersteller (Deklaration) wie auch für die Nachmessung durch Konsumentenschutzorganisationen (Verdikt). Im Gegensatz zu einigen anderen Ländern erfüllen in Deutschland (Stiftung Warentest) und der Schweiz (Kassensturz) viele Sonnenschutzmittel die deklarierten Schutzkriterien. Testresultate sind beispielsweise auf der Homepage der Stiftung Warentest unter Gesundheit und Pressemitteilungen über mehrere Jahre abrufbar. Die große Variabilität stellt ganz generell viele wissenschaftliche Arbeiten infrage, in denen Aussagen und Konklusionen auf möglicherweise ungenauen SPF-Werten basieren.

### Natürliches versus künstliches Sonnenlicht.

Die Beobachtung, dass aufgrund der unterschiedlichen Wirkung von künstlichem und natürlichem Sonnenlicht der SPF auf allen Produkten zu hoch bewertet ist, sollte zu rasch umzusetzenden Konsequenzen in der Deutung und Kommunikation dieses Schutzparameters führen. Alle Produktanbieter sollten umgehend darauf verzichten, den SPF als Multiplikator zusammen mit dem individuellen Hauttyp zur Berechnung der persönlichen Schutzzeit zu verwenden. Die Schutzniveaus niedrig (SPF 6, 10), mittel (SPF 15, 20, 25), hoch (SPF 30, 50) und sehr hoch (SPF 50+) behalten ihre Gültigkeit als Kriterium zur groben Beurteilung der Wirksamkeit [5].

### Erythembelastung.

Um Probanden bei der Messung des SPF_ISO24444_ und des UVA-PF_ISO24442_ nicht potenziell schädlicher Strahlung auszusetzen, wurde eine Reihe von nicht belastenden Verfahren entwickelt.

- Eine erste ISO-normierte In-vitro-Methode ist bereits zur Bestimmung des UVA-Schutzes im Einsatz (UVA-PF_ISO24443_) [19]. Eine weitere In-vitro-Methode liegt derzeit dem technischen ISO-Komitee TC 217 (Kosmetik) als Entwurf vor [21, 24, 25, 28]. Bei diesen Messungen wird die Transmission der Strahlung durch einen Sonnenschutzmittelfilm, der auf einem Substrat aufgetragen ist, gemessen (z. B. aufgeraute PMMA[Polymethylmethacrylat]-Platten).
- Bei der hybriden diffusen Reflexionsspektroskopie (HDRS) handelt es sich um eine kombinierte In-vivo‑/In-vitro-Methode (Abb. 2). Die diffuse Reflexion der Strahlung durch menschliche Haut, die mit dem zu untersuchenden Sonnenschutzmittel bedeckt ist, wird mit einer In-vitro-Transmissionsmessung desselben Produkts auf aufgerauter PMMA-Platte kombiniert. Der In-vivo-Teil führt zum UVA-PF (320–400 nm) und berücksichtigt dabei Wechselwirkungen des Sonnenschutzmittels mit der Haut. Die UVB-Absorption (290–320 nm) wird in vitro gemessen, da Letztere reflexionsspektroskopisch nicht in vivo ermittelt werden kann. Bei dieser Methode werden keine strahlungsbedingten erythemalen Hautreaktionen ausgelöst [30, 31]. Die HDRS-Methode wurde bereits Inter-Labor-Variabilitätsuntersuchungen unterzogen und liegt derzeit dem technischen ISO-Komitee TC 217 (Kosmetik) als Entwurf vor [22]. Diese Methode wird seit 2020 von der Stiftung Warentest zur Überprüfung von SPF-Angaben auf Handelsprodukten verwendet [34]. Ähnliche Verfahren wurden auch von Cole et al. in den USA und von Lademann et al. in Berlin entwickelt [4, 36].
- Anstelle experimenteller Messungen lässt sich die Transmission durch einen Sonnenschutzfilm auch in silico berechnen. Die Berechnung basiert auf den Spektren der verwendeten Filter einschließlich möglichem Abbau derselben, der quantitativen Zusammensetzung und einem Modell des Produktefilms auf der Haut [16]. Die Simulationen sind nicht nur nützlich für die Entwicklung neuer Sonnenschutzmittelformulierungen, sondern tragen auch zum Verständnis von Sonnenschutzmitteln im Allgemeinen bei. Die Simulationswerkzeuge sind im Internet frei verfügbar [1, 11]. Die In-silico-SPF-Berechnung liefert realistische, meist eher konservative Ergebnisse. Solche Berechnungen können auch dazu verwendet werden, experimentell bestimmte SPF-Werte auf ihre Plausibilität zu prüfen oder behördliche Überwachung des Marktes zu realisieren. Voraussetzung sind allerdings Kenntnisse über die quantitative Filterzusammensetzung. Letzteres ist in Ländern, in denen Sonnenschutzmittel als Kosmetika reguliert sind, nicht offengelegt und muss deshalb vorgängig bestimmt werden [10].

**Figure 3:**
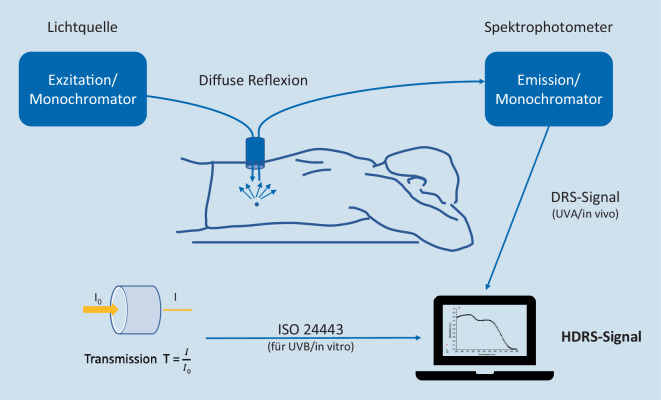

## Quo vadis?

Wie hier dargestellt, ist der SPF_ISO24444_ als Surrogat zur Charakterisierung der wahren Sonnenschutzleistung nur bedingt geeignet. Neben der Zwischen-Labor-Variabilität und der Erythembelastung ist es v. a. die unterschiedliche biologische Wirkung des natürlichen und künstlichen Sonnenlichts, die Fragen aufwirft (Abb. 1; [8]). Die unterschiedliche Wirkung wurde von Diffey und Osterwalder theoretisch diskutiert und mit experimentellen Daten aus Humanversuchen von Hughes und anderen untermauert [2, 8, 14, 15, 17, 23]. Hughes’ Gruppe exponierte Probanden natürlichem Sonnenlicht, deren ausgesparte Hautstellen auf dem Rücken mit unterschiedlichen Sonnenschutzmitteln bedeckt waren (Abb. 2; [17]). Die Expositionszeit war bei allen Probanden gleich. Das Surrogat war die Anzahl Hautstellen in Prozent, die Erytheme aufwiesen. Bei 2 Produkten (G1) waren rund 80 %, bei 5 Produkten (G2) waren knapp 30 % und bei 2 Produkten (G3) waren rund 10 % der Hautareale erythematös. Der Stoff der Schutzkleidung (G4), unter dem kein Erythem entstehen konnte, diente bei jedem Probanden quasi als Negativkontrolle. Die beiden Sonnenschutzmittel mit SPF_FDA_ 30, 45 (G1) zeigten gegenüber den Sonnenschutzmitteln mit SPF_FDA_ 50–110 (G2) einen deutlich geringeren Schutz. Die beiden Sonnenschutzmittel mit SPF_ISO24444_ 40, 45 (G3), die Breitspektrum-UV-Filter enthielten, zeigten klar den besten Schutz im Vergleich zu den vorgenannten Produktgruppen (G1, G2). Das Experiment zeigt darüber hinaus, dass nicht nur die Höhe des SPF-Wertes entscheidend für den umfassenden Schutz ist, sondern auch die breite Schutzwirkung eines Produkts durch anwesende Breitspektrum-UV-Filter (G3). Der beste Schutz wird durch die Kleidung (G4) erreicht, wodurch das gesamte Spektrum des Lichts gleichmäßig reduziert wird (entsprechend dem angelsächsischen Schutzslogan „cover up“). Diese ideale Situation hat den Begriff der spektralen Homöostase geprägt [12]. Granger et al. haben ebenfalls Sonnenschutzmittel unter natürlichem Sonnenlicht untersucht und stellen mithilfe von klinischen Erythema- und Pigmentierungsscores Unterschiede zwischen Produkten mit unterschiedlichen SPFs fest [14, 15]. Die Untersuchungen mit natürlichem Sonnenlicht sind wichtig für ein umfassendes Verständnis des Sonnenschutzes. Für Routineuntersuchungen sind sie allerdings wenig geeignet.

SPF-Wert und Schutzbreite sind entscheidend für umfassenden Schutz

Zur Erfassung eines optimalen Sonnenschutzes ist es notwendig, die Schutzwirkung eines Produkts über einen möglichst weiten Bereich des Spektrums des natürlichen Sonnenlichts zu charakterisieren. Die beiden In-vivo-Methoden SPF_ISO24444_ und UVA-PF_ISO24442_ liefern nur Schutzinformationen zu einem begrenzten Bereich des Spektrums (normierte Lampen und Filter) und ignorieren die Wirkung des nicht berücksichtigten Bereichs des Spektrums. Dies ist eindrücklich in Abb. 2 dargestellt (G1 und G3). Produkte mit gleichen oder ähnlichen SPFs können durchaus unterschiedliche Schutzleistungen liefern. Mit den oben beschriebenen alternativen Methoden (in vitro, in vitro/in vivo und in silico) lassen sich Schutzinformationen zum Spektrum des natürlichen Sonnenlichts, die auf der Absorption, Reflexion und Streuung beruhen, messen und berechnen. Das heißt, der Schutz wird monochromatisch – Wellenlänge für Wellenlänge – erfasst und zeigt auf, inwieweit das Schutzprofil eines Sonnenschutzmittels dem optimalen Schutzprofil der spektralen Homöostase entspricht [7, 9].

Eine breite und routinemäßige Anwendung der alternativen Methoden zur Produktkennzeichnung im Markt steht noch aus. Ein vom technischen ISO-Komitee ISO/TC 217 initiiertes Konsortium ALT-SPF ist zurzeit an der Erarbeitung von umfassenden Lösungen, um die alternativen Bestimmungsverfahren einzeln und in Kombination auf deren Eignung zur Leistungscharakterisierung von Sonnenschutzmitteln prüfen [6].

Bei der Leistungscharakterisierung von Sonnenschutzmitteln stehen wir heute an einem entscheidenden Punkt, mit potenziell geeigneten In-vitro‑, In-vitro‑/In-vivo- und In-silico-Methoden Schutzinformationen wesentlich zu verbessern und uns vom weltweiten Wettbewerb der immer höheren SPF-Werte (v. a. außerhalb Europas) zu verabschieden. Die Rückbesinnung auf die für alle verständliche Beschreibung *niedriger*, *mittlerer*, *hoher* und *sehr hoher* Schutz, der das Spektrum des natürlichen Sonnenlichtes einschließt (und nicht nur die selektiven Bereiche des SPF_ISO24444_ und UVA-PF_ISO24442_), schließt den Kreis zu den ersten Messungen von Rudolf Schulze in den Alpen unter freiem Himmel.

## Fazit für die Praxis

- Die mit künstlichem Sonnenlicht bestimmten SPF-Werte (wie auf allen Sonnenschutzmitteln abgebildet) sind im Vergleich zur SPF-Bestimmung mit natürlichem Sonnenlicht zu hoch. Es empfiehlt sich generell, nur Produkte mit hohen bzw. sehr hohen SPF Werten zu empfehlen.
- Die SPF-Messung ist mit großer Interlaborvariabilität behaftet. Die Charakterisierung eines Sonnenschutzmittels durch den Hersteller wie auch die Nachmessung durch Konsumentenschutzorganisationen sollten sich auf Messungen von mehreren Laboren stützen.
- Der SPF-Wert allein beschreibt den Schutzcharakter eines Produkts unvollständig. Ein Schutz im UVA-Bereich wird durch Breitspektrum UV-Filter erreicht. Für den Konsumenten ist dieser Schutz durch das UVA Logo erkennbar.
- Im Gegensatz zur klassischen SPF-Bestimmung sind die in der Entwicklung befindlichen alternativen Methode nicht-invasiv und liefern Schutzinformationen über einen großen Teil des Sonnenlichtspektrums.
